# A Small Molecule β2 Integrin Agonist Improves Chronic Kidney Allograft Survival by Reducing Leukocyte Recruitment and Accompanying Vasculopathy

**DOI:** 10.3389/fmed.2014.00045

**Published:** 2014-11-12

**Authors:** Samia Q. Khan, Lingling Guo, David J. Cimbaluk, Hatem Elshabrawy, Mohd Hafeez Faridi, Meenakshi Jolly, James F. George, Anupam Agarwal, Vineet Gupta

**Affiliations:** ^1^Department of Internal Medicine, Rush University Medical Center, Chicago, IL, USA; ^2^Department of Surgery, University of Alabama at Birmingham, Birmingham, AL, USA; ^3^George M. O’Brien Kidney Research Center, University of Alabama at Birmingham, Birmingham, AL, USA; ^4^Department of Pathology, Rush University Medical Center, Chicago, IL, USA; ^5^Department of Medicine, Division of Nephrology, University of Alabama at Birmingham, Birmingham, AL, USA

**Keywords:** allograft, macrophage, inflammation, leukadherin, chronic rejection

## Abstract

Kidney allograft rejection is associated with infiltration of inflammatory CD11b+ leukocytes. A CD11b agonist leukadherin-1 (LA1) increases leukocyte adhesion, preventing their transmigration and tissue recruitment *in vivo*. Here, we test the extent to which LA1-mediated activation of CD11b/CD18 enhances kidney allograft survival in a mouse model of fully MHC-mismatched orthotopic kidney transplantation, where C57BL/6J (H-2^b^) recipients received kidney allografts from Balb/c mice (H-2^d^). Isograft control recipients received a kidney from a littermate. Control isograft and allograft recipients were treated daily with cyclosporine (CsA) for 2 weeks, while the test group received CsA therapy and daily LA1 injections during week 1 and alternate days during weeks 2–8. LA1 treatment reduced interstitial leukocyte infiltration in the allograft, reduced neointimal hyperplasia and glomerular damage, and prolonged graft survival from 48.5% (CsA only) to 100% (CsA and LA1) on day 60. Serum creatinine levels showed significantly improved kidney function in LA1-treated mice compared to CsA-treated allograft controls [0.52 ± 0.18 mg/dL vs 0.24 ± 0.07 mg/dL (*n* = 5), respectively]. Furthermore, combination therapy reduced macrophage infiltration and increased the frequency of FoxP3 + Tregs in the allograft. These findings indicate a crucial role for CD11b/CD18 in the control of leukocyte migration to the transplanted kidney and identify integrin agonist LA1 as a novel potential therapeutic agent for kidney transplantation.

## Introduction

Kidney transplantation is the treatment of choice for majority of patients with end stage renal disease (ESRD) and provides a significant survival advantage over chronic dialysis (1). Success rates of transplantation and engraftment of kidney allografts in patients have also significantly improved in the last several decades, led by major scientific and technical advancements in the field. Newer immunosuppressive drugs that efficiently target the adaptive immune system have dramatically reduced the incidence of acute rejection, providing improved quality of life and survival for patients. However, long-term graft survival rates have not changed, with mean half-life of kidney allografts from cadaveric donors currently still at <9 years (2). Kidney allograft failure accounts for >25% of patients with ESRD and prior allograft recipients currently represent one in five kidney transplants performed in the US (3–7). The leading cause of kidney allograft failure after the first year is a clinicopathologic finding often referred to as chronic allograft nephropathy (8), which develops in a majority of kidney allograft recipients. Chronic allograft nephropathy is characterized by a progressive loss of kidney function that is associated with pathologic changes in the kidney glomeruli, the blood vessels, interstitium, and the tubules. Additionally, chronic vascular inflammation in the allograft results in neointima formation and narrowing of the lumina, leading to vasculopathy, also a significant long-term complication of the transplantation (9).

It was recognized early that T-cells and the adaptive immune system play a critical role in allograft rejection, which resulted in the development of remarkably effective immunosuppressive agents for use in various transplantation procedures (10). In addition, cells of the innate immune system, such as macrophages, have also been recognized for their role in promoting acute rejection (11–16). The level of macrophage infiltrate in patient grafts is predictive of kidney allograft survival and the development of chronic allograft nephropathy (11) and it also predominates in renal vascular rejection (17, 18). Recent studies also show that, by directly recognizing and damaging allografts, the innate immune cells play a role in chronic rejection as well (19) and, thus, serve as alternative therapeutic targets to overcome acute and chronic rejection (20). Interstitial macrophage infiltration has been shown to promote acute kidney allograft rejection even in the setting of aggressive T-cell depletion therapies (21, 22). Macrophages promote smooth muscle and endothelial cell injury that provoke vascular damage resulting in intimal proliferation and luminal occlusion and ultimately allograft rejection (23). Moreover, macrophages infiltrate thickened intima of renal arteries (24, 25) and cardiac allografts (26) and contribute to transplant vasculopathy, further contributing to chronic rejection. Indeed, macrophage depletion reduces kidney allograft rejection (12, 27) and the development of cardiac vasculopathy post-transplantation (26). Antibody-mediated blocking or genetic ablation of macrophages decreases intimal thickening in experimental models of inflammatory injury (28). These data suggest that targeting of inflammatory macrophages may provide significant therapeutic benefit in kidney allograft transplantation. However, there are few effective agents available in the clinic that directly targets these cells.

The β2 integrin CD11b/CD18 (also known as Mac-1 and CR3) is expressed in an inactive conformation primarily in innate immune cells including macrophages, neutrophils, and dendritic cells (DCs) (29–32). Upon inflammatory stimuli (33–35), CD11b/CD18 is rapidly activated via a conformational switch to mediate leukocyte migration from circulation to the inflamed tissue by binding to ICAM-1 (CD54) (36–38) on the surface of vascular endothelial cells that is also upregulated in injury (39). CD11b promotes allograft rejection (40) and targeting CD11b/CD18 or its ligand ICAM1 with antagonists that block the adhesion of leukocytes to endothelial cell surfaces reduces inflammatory injury (28, 41, 42), suggesting that the integrin CD11b/CD18 is an important innate immune cell target for developing novel therapeutics against inflammation and transplant rejection. However, antagonists have had limited success in treating inflammatory or autoimmune diseases in patients (43, 44). We recently reported an alternative therapeutic approach for mitigating inflammation that involves the activation, rather than inhibition, of CD11b/CD18-dependent cell adhesion using novel small molecule agonists. Our CD11b/CD18 agonists enhance cell adhesion and significantly reduce leukocyte migration into the inflamed tissue resulting in significant decrease in inflammatory injury in multiple experimental models (45–47).

In the present study, we utilized our lead CD11b/CD18 agonist compound leukadherin-1 (LA1) to test whether LA1 treatment would also decrease macrophage infiltration in kidney allografts and whether that would result in a functional benefit, such as decreased neointimal hyperplasia, enhanced kidney function, and prolonged allograft survival. We utilized a recently described murine model of kidney allograft transplantation using fully MHC-mismatched orthotopic kidney transplant, in which C57BL/6J (H-2^b^) recipients received kidney allografts from Balb/c mice (H-2^d^) that closely mimics clinicopathologic findings of human kidney transplants (9), and compared the efficacy of standard immunosuppressive cyclosporine (CsA) therapy with that of CsA in combination with LA1 for up to 8 weeks. These findings identify the CD11b/CD18 agonist LA1 as a novel therapeutic agent for improving kidney transplantation.

## Results

### Rejected human kidney allografts show increased CD11b+ leukocyte infiltration

**Figure 1:**
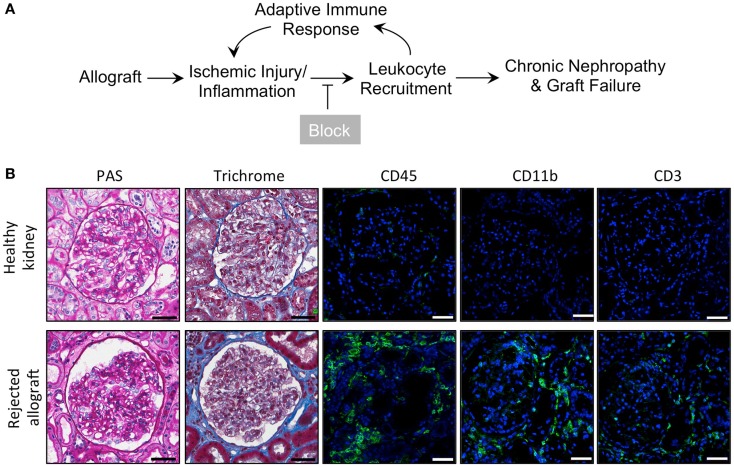
**Inflammatory leukocyte infiltrates are present in rejected human allografts**. **(A)** Schematic diagram illustrating our working model. In response to ischemic kidney injury or inflammation upon allograft transplantation, CD11b+ leukocytes are recruited into the tissue, where they induce an adaptive response and further recruitment of innate and adaptive immune cells. Increased inflammation results in progressive tissue damage, nephropathy and, eventually, graft failure. Given that CD11b+ leukocyte infiltration into the allograft is an early event, blocking influx of these innate immune cells via novel small molecule CD11b agonists is expected to reduce inflammation, decrease progressive tissue damage and prolong allograft survival. **(B)** Representative micrographs of human kidney tissue sections (from a healthy cadaveric donor or a rejected allograft) after histochemical and immunofluorescence analyses. Sequential sections from each tissue were stained with periodic acid-Schiff (PAS) or trichrome and imaged using light microscopy. Sections were also stained with antibodies specific for CD45, CD11b, or CD3, as indicated, followed by fluorescently labeled secondary antibodies and then imaged using a confocal microscope. The rejected allograft shows enhanced trichrome staining (indicating an increase in interstitial fibrosis) and increased numbers of CD45+, CD11b+, and CD3+ immune cells as compared to the healthy kidney (*n* = 4–5/group). Scale bar = 50 μm.

Kidney allograft transplantation leads to an influx of a variety of leukocytes into the graft. Macrophages account for the majority of infiltrating leukocytes in acute rejections (48). We hypothesize that pharmacological blockade of macrophage recruitment to the allograft using our novel CD11b agonists (45) will prolong allograft survival and enhance allograft function (Figure 1A). To confirm that rejected human kidney allografts contain a high number of CD11b-expressing leukocytes (48), which could be targeted with LA1, we used fluorescence confocal microscopy to analyze biopsy tissue from healthy patient kidneys and from rejected allografts stained with antibodies specific for CD45 (a pan-leukocyte marker), CD11b, and CD3 (Figure 1B). We observed higher numbers of CD45+, CD11b+, and CD3+ cells within the glomeruli and interstitium of the rejected allografts, while leukocytes were absent in the healthy kidneys. Additionally, enhanced trichrome staining in the rejected allograft tissue suggested increased fibrosis (Figure 1B).

### LA1 treatment, in combination with CsA, prolongs allograft survival and preserves kidney function

**Figure 2:**
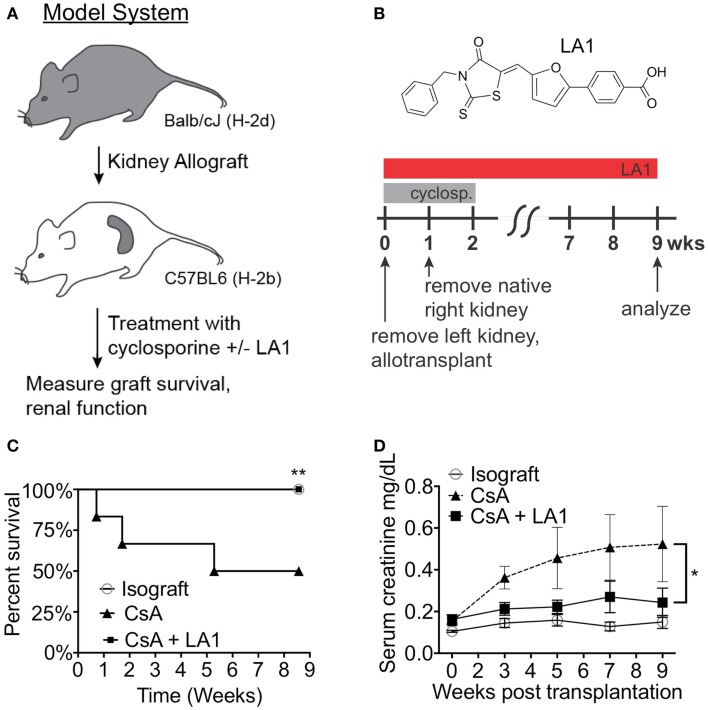
**LA1 treatment prolongs transplant survival and increases kidney function**. **(A)** A flow diagram describing the mouse model of chronic allograft nephropathy utilized in this study. **(B)** Schematic representing the timeline of treatment with CsA and/or LA1 and the allogeneic kidney transplant surgery as described in the Section “Concise Methods.” The chemical structure of LA1 is also shown on the top. **(C)** A Kaplan–Meier plot of graft survival for isografts (non-filled circles, *n* = 3) or allografts treated with either CsA alone (triangles, *n* = 4) or a combination of CsA and LA-1 (squares, *n* = 5) over time (in weeks). Significance was determined using the Log-rank (Mantel–Cox) test. **P < 0.01 **(D)** A plot showing LC-MS/MS-based quantification of serum creatinine levels in various samples at the indicated time points. Whole blood was collected at the indicated time points via retro-orbital puncture from experimental animals from isograft group (non-filled circles) or allograft groups treated with either CsA alone (triangles) or a combination of CsA and LA-1 (squares). Data shown are mean ± SEM (*n* = 5/group). Significance was determined using a two-tailed Student’s *t*-test and the calculated *P*-value is shown. *P < 0.05.

LA1 administration reduces leukocyte recruitment and kidney injury in a model of anti-GBM nephritis (45). Furthermore, LA1 treatment provided significant protection of WT B6 mice from renal ischemia-reperfusion injury (IRI) (Figure S1 in Supplementary Material). These data suggest that LA1 has significant reno-protective effects in an acute setting. Next, we investigated the efficacy of LA1 on kidney allograft function and survival. As previously described (9), the left kidney in the recipient C57BL/6J animal was removed and was replaced with a donor Balb/c kidney and the animals (*n* = 4-5 per group) were treated with CsA for 2 weeks post transplantation to prevent acute allograft rejection (Figures 2A,B). The native right kidney was removed 1 week later. Kidney function and graft survival was monitored in animals for up to 8 weeks post-transplantation, as described in the Section “Concise Methods.” One group of animals (LA1 group) were treated *intraperitoneally* (*i.p*.) with LA1 (2.5 mg/kg) daily for 1 week and every other day for the remaining weeks until the end of the study (2–8 weeks post-transplantation). The isograft control group comprised C57BL/6J (H-2b) recipients that received a kidney from a C57BL/6J (H-2b) littermate. Recipient survival is significantly reduced in this model, with a measureable decline in kidney function beginning at 3 weeks post-transplantation (9). The results show that approximately 50% of CsA-treated animals were lost during the 8 weeks of the experiment due to loss of the transplanted allograft, whereas all animals with isografts survived (Figure 2C). Surprisingly, CsA + LA1-treated animals showed 100% survival and graft protection. Serum creatinine levels indicated significantly improved kidney function in LA1-treated mice compared to allograft controls treated with CsA (0.52 ± 0.18 mg/dL of creatinine for CsA group vs 0.24 ± 0.07 mg/dL for CsA + LA1 group at end-point, Figure 2D). Similarly, urinary analysis showed a much higher level of proteinuria in the CsA only treated animals versus the CsA + LA1 group (11.7 ± 6.6 mg/mg of albumin/creatinine for CsA group vs 3.6 ± 0.4 mg/mg for CsA + LA1 group at end-point).

**Figure 3:**
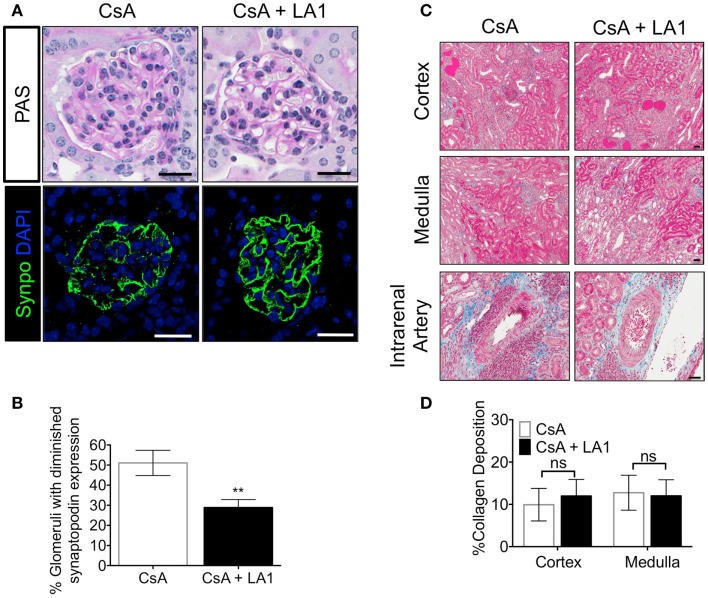
**LA1 treatment protects glomerular synaptopodin expression but does not reduce interstitial fibrosis in the allograft**. **(A)** Representative images from allograft kidney sections of control (CsA alone) and LA1-treated (CsA + LA1) animals (upper panels) showing glomeruli after staining tissue sections with PAS and imaged using light microscopy or (lower panels) after staining with an antibody against the podocyte marker synaptopodin (synpo, green) [nuclei were stained with DAPI (blue)] and imaged using a fluorescence microscope. Images are representative of five independent samples per group. Scale bar = 25 μm. **(B)** A plot showing quantification of synpo staining in the allograft glomeruli as shown in **(A)** (as percent glomeruli that showed diminished synpo staining per tissue section) from animals treated either with CsA or with a combination of CsA and LA-1. Data shown are mean ± SEM (*n* = 5/group). Significance was determined using a two-tailed Student’s *t*-test. **P < 0.01. **(C)** Representative images from allograft kidney sections of control (CsA alone) and LA1-treated (CsA + LA1) animals showing the cortex, medulla, and the intrarenal arteries, as labeled. Images are representative of 4–5 independent samples per group. Scale bar = 50 μm. **(D)** A plot showing quantification of collagen deposition in whole trichrome-stained kidney sections as shown in **(C)** and analyzed using an Aperio ScanScope (shown as percent collagen staining per tissue section) from animals treated either with CsA alone or with a combination of CsA and LA-1. Data shown are mean ± SEM (*n* = 4–5/group). Significance was determined using a two-tailed Student’s *t*-test. ns: not statistically significant.

Downregulation of synaptopodin (synpo), a hallmark of differentiated podocytes, has been associated with proteinurea and serves as a prognostic indicator of human glomerulopathies (49, 50). Next, to investigate if the improved kidney function in CsA + LA1 group is associated with reduction in damage to the glomeruli, we stained kidney sections from the two groups of animals with an antibody against synpo and analyzed the expression using confocal fluorescence microscopy. We found that the allografts treated with CsA showed remarkably weaker expression of Synpo as compared to the CsA + LA1-treated allografts (Figures 3A,B), suggesting that LA1 administration protected glomeruli from damage. We also analyzed the kidney sections using trichrome staining to study any changes in the level of fibrosis (Figure 3C). Quantitative analysis revealed similar amounts of interstitial collagen deposition of approximately 10–15% of the medulla and cortex in both the LA1 treated and control allografts (Figure 3D), suggesting that LA1 treatment did not significantly reduce the level of fibrosis in this study. Given that the study period was only 8 weeks, it is possible that this time frame is not long enough to provide information on interstitial fibrosis, which might require longer, future studies to determine if LA1 treatment can also affect fibrosis. Overall, the results presented here confirm that mismatched kidney allograft recipients that are treated with LA1 have improved kidney function and prolonged allograft survival as compared to CsA only treated allograft controls.

### LA1 treatment reduces leukocyte infiltration and decreases neointimal hyperplasia

**Figure 4:**
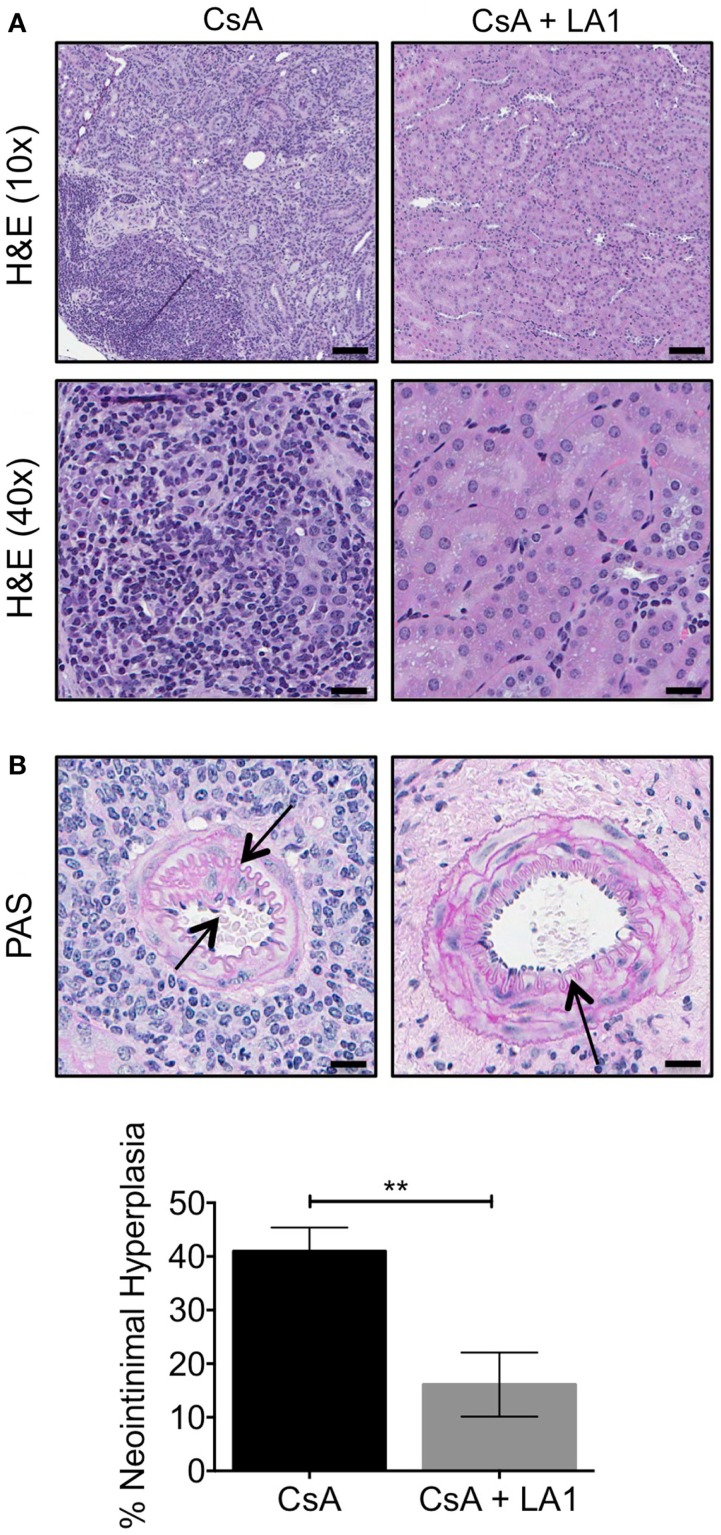
**LA1 treatment reduces leukocyte infiltrate and neointimal hyperplasia**. **(A)** Representative H&E stained micrographs of allograft kidney tissue sections from control (CsA alone) and LA1-treated (CsA + LA1) animals showing significant reduction of mononuclear cell infiltrates with LA1 treatment. Images are representative of five independent samples per group. Scale bar = 100 μm (in the H&E 10× panels) and 25 μm (in the H&E 40× panels). **(B)** Representative PAS-stained micrographs of allograft kidney tissue sections from control (CsA alone) and LA1-treated (CsA + LA1) animals showing extensive perivascular leukocyte infiltration and intrarenal arterial neointimal hyperplasia in CsA-treated mice that is significantly reduced with LA1 co-treatment. Images are representative of five independent samples per group. Scale bar = 25 μm. Below, a plot showing quantification of neointimal hyperplasia in the intrarenal arteries, as shown in **(B)** and quantified as described in the Section “Concise Methods,” from animals treated either with CsA alone or with a combination of CsA and LA1. Data shown are mean ± SEM (*n* = 5/group). Significance was determined using a two-tailed Student’s *t*-test. **P < 0.01.

We previously reported that this murine model shows striking resemblance to the intrarenal vasculopathy characterized by perivascular leukocytic infiltration and neointimal hyperplasia that is observed in rejected patient allografts. Here, we analyzed kidney sections from allograft recipients to assess the effect of LA1 treatment on leukocyte infiltration and neointimal hyperplasia. Hematoxylin-eosin (H&E) staining of graft tissue sections revealed extensive infiltration of leukocytes in kidney tissue of the CsA-treated allograft controls (Figure 4A), which was drastically reduced with LA1 treatment. Importantly, periodic acid-Schiff (PAS) histological analysis revealed that LA1-treated allograft recipients developed reduced perivascular leukocyte infiltration and reduced neointimal hyperplasia, which are both manifestations of transplant arteriosclerosis (9). Quantitative analysis for neointimal hyperplasia in intrarenal arteries revealed that the allograft recipients treated with CsA alone had significantly higher level of neointimal hyperplasia as compared to LA1-treated animals, which were comparable to isograft controls (9) {40.98 ± 4.41% for CsA group vs 16.11 ± 5.99% for CsA + LA1 group [*P*-value = 0.001 (*n* = 5)], Figure 4B}. This suggests that LA1 treatment reduces vascular injury in the setting of kidney allografts.

### Administration of LA1 reduces infiltrating macrophages within the allograft and boosts number of Foxp3+ regulatory T-cells in the graft

**Figure 5:**
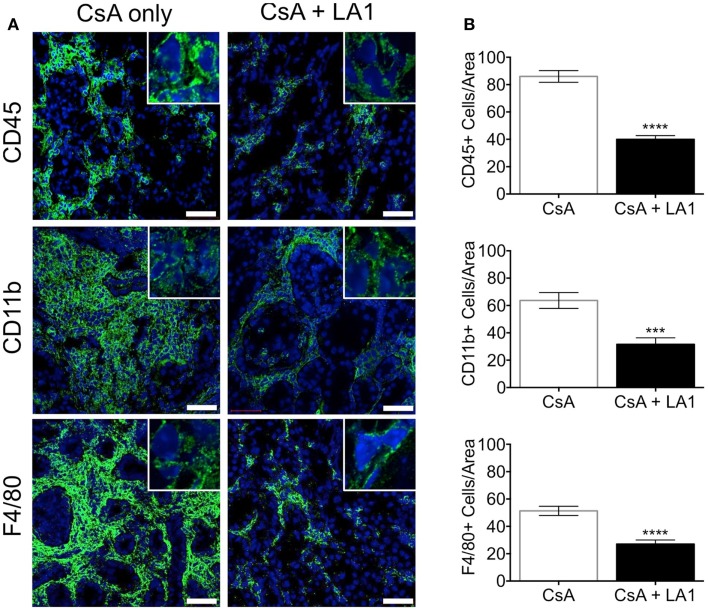
**LA1 treatment reduces perivascular infiltration of macrophages**. **(A)** Representative micrographs of kidney allograft tissue sections from control (CsA alone) and LA1-treated (CsA + LA1) animals after immunofluorescence analyses. Sequential sections from each tissue were stained with antibodies against CD45, CD11b, or F4/80, as indicated, followed by fluorescently labeled secondary antibodies and then imaged using a confocal microscope. Inset shows image of an area of 60× magnification. Images are representative of 4–5 independent samples per group. Scale bar = 50 μm. **(B)** Plots showing quantification of number of positive cells/area for each marker, as shown in **(A)**. Three random areas per tissue were evaluated at 60× magnification by manually counting positively stained cells. Data shown are mean ± SEM (*n* = 4–5/group). Significance was determined using a two-tailed Student’s *t*-test. ***P < 0.001; ****P < 0.0001.

**Figure 6:**
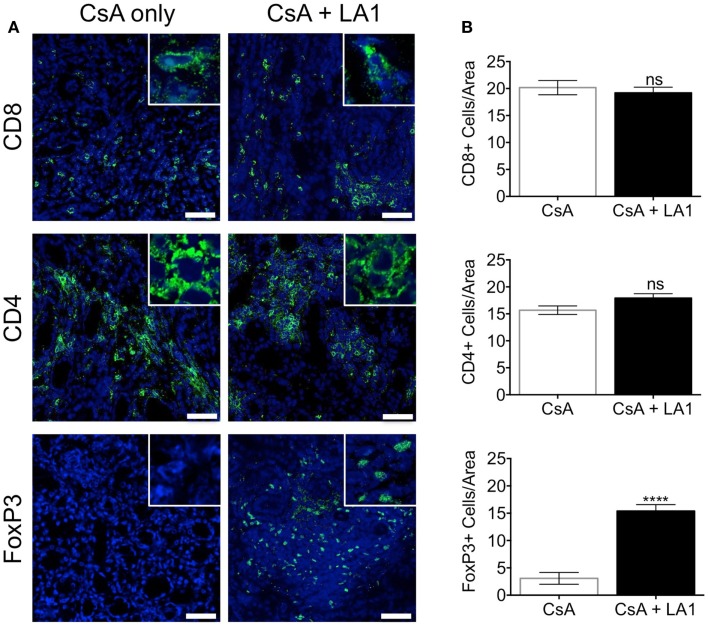
**LA1 treatment does not affect the number of CD8+ and CD4+ T-cells infiltrating the allograft but increases the frequency of infiltrating FoxP3+ regulatory T-cells**. **(A)** Representative micrographs of kidney allograft tissue sections from control (CsA alone) and LA1-treated (CsA + LA1) animals after immunofluorescence analyses. Sequential sections from each tissue were stained with antibodies against CD8, CD4, and intracellular FoxP3 in allografts, as indicated, followed by fluorescently labeled secondary antibodies and imaged using a confocal microscope. Inset shows image of an area at 60× magnification. Images are representative of 4–5 independent samples per group. Scale bar = 50 μm. **(B)** Plots showing quantification of the number of positive cells/area for each marker, as shown in **(A)**. Three random areas per tissue were evaluated at 60× magnification by manually counting positively stained cells. Data shown are mean ± SEM (*n* = 4–5/group). Significance was determined using a two-tailed Student’s *t*-test. ns: not statistically significant. ****P < 0.0001.

LA1 treatment reduces CD11b+ macrophage recruitment to the site of inflammation (45). To test whether the LA1-mediated protection of kidney allografts and the reduction in allograft vascular injury was associated with a decrease in CD11b+ macrophage influx, we quantified the immune cell subsets using immunofluorescence staining and confocal microscopy. Immunofluorescence microscopy revealed that the extensive perivascular leukocytic infiltrate observed in the CsA-treated allografts was mainly composed of CD11b+ macrophages (Figure 5A) (9). LA1 treatment resulted in a significant twofold reduction in total CD45+ leukocytes within the allograft as compared to allograft controls. More importantly, a simultaneous twofold reduction in allograft infiltrating CD11b+ and F4/80+ macrophages was observed in the LA1-treated recipients as compared to control allografts (Figure 5B). Furthermore, immunofluorescence staining and analysis of T-cells showed that LA1 treatment had no effect on the number of CD8+ and CD4+ T-cells infiltrating the allograft (Figures 6A,B).

FoxP3+ regulatory T-cells (Tregs) have been reported to mediate allograft tolerance and are expanded in stable kidney allografts (51–54). Macrophages recruited to the site of inflammation become potent producers of proinflammatory cytokines including IL-1β, which prevents the induction of *de novo* Tregs and also convert existing Tregs into proinflammatory Th17 cells (55–57). To study whether the LA1-mediated reduction of macrophages in the allograft affected the frequency of Tregs in the allograft, we stained and quantified Tregs in the two groups of allograft kidney sections using immunofluorescence. Surprisingly, we found that LA1 treatment resulted in a significant fivefold increase in the number of intrarenal FoxP3+ regulatory T-cells in the allograft tissue as compared with the CsA only treated allografts (Figures 6A,B). These data clearly indicate that LA1 treatment resulted in an anti-inflammatory intrarenal immune cell signature (fewer macrophages and more Tregs) that is indicative of a stable allograft.

## Discussion

Kidney allograft rejection remains an important post-transplantation complication and is in critical need of newer therapeutic approaches. Drug development efforts over the last several decades have solely focused on modulating the adaptive immune system, which plays a critical role in rejection, resulting in several extremely effective anti-rejection therapeutics that are currently in wide use in the clinic. Infiltration of innate immune cells, primarily macrophages, has also been identified in the allograft biopsies of murine and human kidney transplants (13). Decreased graft survival is associated with increased presence of macrophages, while they are extremely rare in stable transplants surviving long term (13), suggesting that targeting of macrophages could also provide additional benefits, especially in the setting of chronic kidney rejection. Yet, effective therapeutics for reducing macrophage influx in allografts are sorely lacking. The leukocytic integrin CD11b/CD18 plays a vital role in the multistep process of infiltration and accumulation of macrophages in the inflamed tissue. During the past several years, studies have focused on reducing leukocyte infiltration to the inflamed tissue by using blocking antibodies that hinder the binding of CD11b/CD18 to their ligands (28, 41, 42) on the vascular wall or by specifically deleting myeloid cell subsets by genetic ablation of CD11b (30) or CD18 (58). Although these approaches have been successful in decreasing the severity of inflammatory responses in several animal models, such blocking reagents have failed in clinical trials (43, 59) and have been withdrawn from the market (60).

In a previous study, we reported an alternative approach of inhibiting leukocyte migration by enhancing the activation of integrin CD11b/CD18 with agonist LA1 (as compared to the use of integrin antagonists in the literature) (45). LA1-mediated integrin activation decreased inflammation in several experimental models and preserved organ function upon injury. Similarly, clinical trials utilizing blocking antibodies in the kidney transplant setting have resulted in a high incidence of lethal infections and relapses as a result of long lasting immunodeficiency (61) with no difference in the incidence or severity of graft rejection (62–64). Unlike antibodies, small molecule leukadherins are orally available and are rapidly cleared which, while potentially requiring more frequent dosing in the patients, offers the flexibility in dosing and tight control over unwanted immunosuppression (45). Additionally, anti-adhesion therapies (when combined with other immune-suppressive drugs) have also been linked with progressive multifocal leukoencephalopathy (PML) in patients, which was another reason for the initial withdrawal of some of these drugs from the market (60). PML is associated with re-activation of the normally latent JC virus in the patients. Some of the anti-integrin agents target integrin α4β1 and block adhesion of α4β1 expressing cells (such as T-cells, hematopoietic stem cells, and immature B-cells) to ligand VCAM-1. The increased incidence of PML in the anti-adhesion therapy treated patients has been postulated to be likely due to increased escape of progenitor and immature cells from their bone marrow niche (where they are held via α4β1:VCAM-1 interaction) into circulation, carrying JC virus with them. Additionally, it has been postulated that T-cells that normally restrict the JC virus, are unable to cross the blood-brain barrier to contain it in the tissue. We believe that our integrin agonist LA1 will have a significantly lower risk of PML in patients for two reasons: (a) because it enhances CD11b/CD18-dependent cell adhesion (thus reducing risk of CD11b+ progenitors escaping into circulation, without affecting other cell types) and (b) because it primarily targets innate immune cells vs T-cells, thus may not affect migration of T-cells to fight-off JC virus, in case of its re-activation in the brain tissue. However, future studies are needed to fully address this issue.

Here, we studied the ability of LA1 to enhance the effects of CsA treatment in a mouse model of kidney transplantation. CsA is a potent immunosuppressive agent often used in transplantation to prevent acute rejection by dampening T-cell responses. Its immunosuppressive action is mediated through the blockade of cytokine production, which, in turn, modulates immune activation and T-cell proliferation (65). We report that LA1, in combination with CsA, significantly prolongs kidney allograft survival and function as compared to treatment with CsA alone. LA1 treatment resulted in a dramatic reduction of interstitial as well as perivascular inflammatory infiltrates and significantly reduced neointimal hyperplasia. These data show that LA1 treatment reduces transplant arteriosclerosis, which is a significant long-term complication of kidney transplantation and predictor of late graft failure (66, 67).

More comprehensive immune cell phenotyping indicated that the allograft leukocytic infiltrate was mainly composed of macrophages as indicated by CD11b+ and F4/80+ immunostaining. We also determined that LA1 treatment did not change the number of CD4+ or CD8+ T-cells infiltrating the allografts. This can be due to the already dampened T-cell responses targeted by the immunosuppressive CsA. There was, however, an increase in the number of FoxP3+ regulatory T-cells infiltrating the LA1 + CsA-treated allograft tissue as compared to CsA-treated controls. The reasons for this observation are unclear. One possible explanation is that the reduction of macrophages in the allograft results in a decrease in proinflammatory cytokines including IL1, IL6, and TNF-α, which have been reported to impair the differentiation and function of FoxP3+ Tregs (68). Indeed, LA1 treatment directly reduces the production of IL1, IL6, and TNF-α by CD11b+ myeloid cells in both *in vitro* and *in vivo* inflammatory settings (unpublished data). Taken together, our results indicate that LA1 treatment, together with standard CsA immunosuppression, alters the balance between CD11b+ inflammatory macrophages (twofold decrease) and regulatory Foxp3+ Tregs (fivefold increase) resulting in an overall immunosuppressive effect. The alteration of the profile of immune cells in the allograft may underlie the efficacy of LA1 in prolonging kidney transplant survival. For example, LA1 treatment might influence the levels of macrophage phenotypes (M1 vs M2) in the renal compartment. Another possibility is that LA1 affects the function of DCs that are key drivers of immune response against allo-antigens, either the DCs that are resident within the kidney and/or those residing within the secondary lymphoid organs. This could be via LA1 reducing the proinflammatory cytokine levels in the tissue (due to reduced number of macrophages being recruited and activated locally) or due to direct effects on DC migration into the tissue and their activation, given that DCs also express integrin CD11b/CD18. We hope that our planned future studies will clarify this mechanism.

Future studies will determine the details of such cellular phenotype modulation by LA1 *in vivo*. Additionally, it has previously been shown that agonist Mn^2+^-mediated activation of CD11b/CD18 on antigen presenting cells inhibits T-cell activation (69). More studies are needed to determine if LA1-mediated pharmacological activation of CD11b/CD18 has a similar effect. Finally, the present study used a dosing regimen that was initiated immediately prior to injury. Whether such pharmacologic modulation of innate immune cells can stabilize, treat, or reverse established disease is an important question that would be addressed in future studies. Similarly, future studies will be needed to define the consequences of withdrawing LA1. It would be interesting to study whether the imparted tolerance can be maintained in such a scenario.

In summary, we have presented here an alternative approach for kidney transplant immunosuppression that involves activation, rather than the blockade of CD11b/CD118, ultimately resulting in the reduction of leukocyte recruitment to the allograft and inflammation. We suggest that targeting integrins with small molecule agonists may improve the long-term outcome of human transplants and may also provide therapeutic benefit in inflammatory and autoimmune diseases.

## Concise Methods

### Human biopsy samples

Nine kidney biopsies were selected with at least seven glomeruli in the block available for sectioning, regardless of diagnosis. Four were native kidneys and five were allografts. All allografts had been diagnosed with chronic allograft nephropathy involving 15–90% of the cortex (average 37.0% cortex ± 30.1%). The average age was 60.7 ± 12.9 years (41–76 years). This study was approved by the Institutional Review Board (IRB) committee at the Rush University Medical Center.

### Animals

Male C57BL/6J (H-2^b^) and Balb/cJ (H-2^d^) mice were purchased from Jackson Laboratories and were housed under pathogen-free conditions in the Animal Facility at either the University of Alabama at Birmingham or at University of Miami Miller School of Medicine. All animal experiments were performed in accordance with the institutional guidelines under Institutional Animal Care and Use Committee approval. Renal IRI model was performed in C57BL/6J animals as previously described (10).

### Orthotopic kidney transplants

Vascularized orthotopic kidney transplants were performed in mice as previously described (9). Three groups were evaluated. In the isograft group, C57BL/6J (H-2^b^) recipients received a kidney from a C57BL/6J (H-2^b^) littermate. In the CsA alone treated allograft group (control), C57BL/6J (H-2^b^) mice received a kidney from an MHC-mismatched Balb/cJ (H-2^d^) mouse. In the experimental CsA + LA1-treated group (test group), C57BL/6J (H-2^b^) mice were pre-injected with 1 mg/kg of LA1 intravenously 2 h before they received a kidney from a completely mismatched Balb/cJ (H-2^d^) mouse. These were mice subsequently received daily *intraperitoneal* injections (2.5 mg/kg) of LA1 during week 1 and every other day during weeks 2–8. Both the isograft and allograft recipients received CsA at a dose of 10 mg/kg daily subcutaneously for 14 days post-transplantation to prevent acute injury.

### Measurement of kidney transplant function

Whole blood was collected through retro-orbital bleeding and urine was collected at the indicated time points to assess transplant function. Serum creatinine levels were measured as previously described using LC-MS/MS (70). Urine protein was measured using an albumin specific sandwich ELISA (Bethyl Laboratories, Montgomery, TX, USA).

### Histologic analysis and immunohistochemistry

Grafts were harvested at 8 weeks post-transplantation or at the time of rejection. One part of the removed kidney was fixed in 10% formalin and embedded in paraffin and another part was immediately frozen in liquid nitrogen. Tissue sections (4 μm) were stained with H&E, PAS, or Masson trichrome. Histological analysis was performed on digital images that were acquired with a whole slide Aperio Scanner (Leica Biosystems, Buffalo Grove, IL, USA) and analyzed using the ImageScope software. The Aperio Scanscope allowed scanning and quantitation of the whole slide using a 20× objective lens. The ImageScope Colocalization Algorithm was used to quantify whole trichrome-stained kidney tissues for collagen deposition. The Blue (color 1) and Red color (color 2) channels were used. The Blue threshold was set at 210 and Red at 180. In order for a pixel to be considered fibrotic, it had to meet the threshold for Blue but not for Red (% area blue/total area). Neointimal hyperplasia in intrarenal arteries was calculated as described previously (9) using the formula [(neointimal hyperplasia area − lumen area)/neointimal hyperplasia area] × 100 and expressed as a percentage.

### Immunofluorescence staining and confocal microscopy

For frozen sections, human or mouse kidney tissue was embedded in OCT, snap frozen in liquid nitrogen and stored at −80°C. Tissue sections were cut and fixed in −20°C acetone before immunofluorescence staining. Sections were blocked at room temperature for 1 h and incubated with the primary antibodies – CD45 (anti-mouse: Clone 30-F11, 550539,BD Pharmingen, anti-human: Clone HI30, 14-0459-82, eBioscience), CD11b (anti-mouse and anti-human: Clone M1/70, 101202, Biolegend), CD4 (Clone RM4-5, 5505319, BD Pharmingen), CD8 (Clone YTS.169.4, MBS520216, Mybiosource), F4/80 (Clone CI:A3-1, MCA497GA, AbD Serotec), CD3 (anti-human: Clone OKT3, 70-0037, Tonbo Biosciences), WT1 (polyclonal rabbit, SC-192, Santa Cruz), and synaptopodin (polyclonal goat, SC-21537, Santa Cruz) in blocking buffer at 4°C overnight. Sections were incubated with the appropriate secondary antibody (Invitrogen Life Technologies, Grand Island, NY, USA) and mounted with medium containing DAPI to visualize nuclei (Vector Laboratories, Burlingame, CA, USA). Fluorescence images were acquired using a Zeiss 700 LSM confocal microscope with an iLCI Plan Neofluar 63×/1.3 Oil Imm Corr M27 with a AxioCam camera and analyzed using the Zen software (Carl Zeiss Group, Hartford, CT, USA).

### Statistical analysis

All statistical analyses were performed using Graphpad Prism 6. Data are expressed as means ± SEM. The two-tailed Student’s *t*-test was used for comparison between two groups. Kaplan–Meier survival curves were analyzed with the log-rank (Mantel–Cox) test. *P* values < 0.05 were considered statistically significant.

## Conflict of Interest Statement

Vineet Gupta is an inventor on pending patent applications related to this study. He is also a co-founder of Adhaere Pharmaceuticals, Inc. that has licensed these applications. This author has the potential for financial benefit from their future commercialization. The other co-authors declare that the research was conducted in the absence of any commercial or financial relationships that could be construed as a potential conflict of interest.

## Supplementary Material

The Supplementary Material for this article can be found online at http://www.frontiersin.org/Journal/10.3389/fmed.2014.00045/abstract
