# Supplementary material for: A Small Molecule β2 Integrin Agonist Improves Chronic Kidney Allograft Survival by Reducing Leukocyte Recruitment and Accompanying Vasculopathy
Source: Front Med (Lausanne). 2014 Nov 12;1:45. doi: 10.3389/fmed.2014.00045 (PMC4291902; doi:10.3389/fmed.2014.00045)
Supplement: Supplementary file 1 [file Data_Sheet_1.PDF]

Supplementary Information

Supplementary Figure

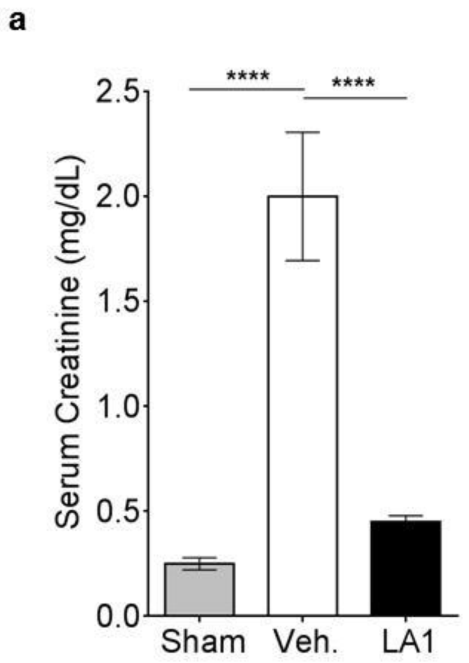

Figure S1. LA1 protects mice from renal ischemia-reperfusion injury (IRI). **a-b.** Graphs showing effects of LA1 administration on renal function after IRI. Kidneys of WT C57BL/6J male mice (8 - 12 weeks of age) were subjected to 30 min of ischemia and 24 h reperfusion. Sham treated animals underwent identical surgery but without application of the clamps. Animals were administered LA1 (1mg/kg) or vehicle (DMSO) in saline intravenously 30 min prior to surgery and renal IR injury was assessed at 24 h post-ischemia by measuring the serum creatinine (sCr) and blood urea nitrogen (BUN) levels. Graphs showing sCr levels (a) and BUN levels (b) in mice that underwent sham surgery (n=3) or IRI and were treated with vehicle (n=4) or LA1 (n=4) are presented. Data shown are means ± SEM. **** $p < 0.0001$.
